# Multilocus Sequence Subtyping and Genetic Structure of *Cryptosporidium muris* and *Cryptosporidium andersoni*

**DOI:** 10.1371/journal.pone.0043782

**Published:** 2012-08-24

**Authors:** Rongjun Wang, Fuchun Jian, Longxian Zhang, Changshen Ning, Aiqin Liu, Jinfeng Zhao, Yaoyu Feng, Meng Qi, Helei Wang, Chaochao Lv, Guanghui Zhao, Lihua Xiao

**Affiliations:** 1 College of Animal Science and Veterinary Medicine, Henan Agricultural University, Zhengzhou, China; 2 Department of Parasitology, Harbin Medical University, Harbin, China; 3 School of Resource and Environmental Engineering, East China University of Science and Technology, Shanghai, China; 4 College of Veterinary Medicine, Northwest A & F University, Yangling, China; 5 Division of Foodborne, Waterborne, and Environmental Diseases, National Center for Emerging and Zoonotic Infectious Diseases, Centers for Disease Control and Prevention, Atlanta, Georgia, United States of America; Royal Tropical Institute, The Netherlands

## Abstract

In this study, nine *C. muris* and 43 *C. andersoni* isolates from various animals in China were subtyped by a multilocus sequence typing (MLST) tool. DNA sequence analyses showed the presence of 1–2 subtypes of *C. muris* and 2–6 subtypes of *C. andersoni* at each of the four loci (MS1, MS2, MS3, and MS16), nine of which represented new subtypes. Altogether, two *C. muris* and 10 *C. andersoni* MLST subtypes were detected. Linkage disequilibrium analysis indicated although the overall population structure of the two parasites was clonal, the Chinese *C. andersoni* in cattle has an epidemic structure. Three and two clusters were produced in the *C. muris* and *C. andersoni* populations by Structure 2.3.3 analysis, with Chinese *C. muris* and *C. andersoni* substructures differing from other countries. Thus, this study suggested the prevalence of *C. andersoni* in China is not attributed to the introduction of dairy cattle. More studies involving more genetic loci and systematic sampling are needed to better elucidate the population genetic structure of *C. muris* and *C. andersoni* in the world and the genetic basis for the difference in host specificity among the two most common gastric parasites.

## Introduction

*Cryptosporidium muris* was first identified in the gastric glands of mice, but has been shown since to have a wide range of hosts, including various rodents, pigs, bactrian camels, giraffes, dogs, cats, cynomolgus monkeys, seals, bilbies, and birds [1]–[14]. In contrast, *Cryptosporidium andersoni* was long considered *C. muris* and was established as a new species only based on genetic and host specificity differences [15]. Results of studies conducted in numerous countries suggested that *C. andersoni* is mostly a parasite of cattle, only occasionally being detected in other animals such as bactrian camels, sheep, goats, and hamsters [7], [16], [17]. Both *C. muris* and *C. andersoni* are considered minor zoonotic *Cryptosporidium* species based on the fact that a few human cases have been reported in recent years [18]–[28].

Various subtyping tools have been developed for *Cryptosporidium parvum* and *Cryptosporidium hominis* using polymorphic microsatellite and minisatellite markers identified in recent whole genome sequencing data. They have been very useful in molecular epidemiologic and population genetic studies [29]. However, most of these tools can only subtype *C. parvum* and *C. hominis*, two intestinal species of the most public health significance [29], [30]. The recent whole genome sequencing of *C. muris* has allowed the identification of microsatellite and minisatellite markers for gastric *Cryptosporidium* spp. Thus, Feng et al. screened the *C. muris* genome sequence data for microsatellite and minisatellite targets, and developed a multilocus sequence typing (MLST) tool for *C. muris* and *C. andersoni* [31].

The characterization of *Cryptosporidium* genetic structure has direct implications in understanding its biology as well as transmission dynamics and infection sources in different hosts and geographic areas [30]. Previously, population genetic structure analysis was only conducted in *C. parvum* and *C. hominis* and three types of populations were identified, including panmictic populations, clonal populations, and epidemic populations 32–34. The aim of the present study was to subtype *C. muris* and *C. andersoni* isolates and explore the population genetic structure of *C. muris* and *C. andersoni* by mining the MLST data using cluster analysis, diversity statistical test, and measurements of linkage disequilibrium.

## Materials and Methods

### Ethics Statement

This study was performed in accordance with the recommendations in the Guide for the Care and Use of Laboratory Animals of the Ministry of Health, China. Prior to experiment, the protocol of the current study was reviewed and approved by the Research Ethics Committee of Henan Agricultural University. The fecal samples were obtained by the collection of feces excreted from animals after the permission of farm owners, with no specific permits being required by the authority for the feces collection.

### *Cryptosporidium* Isolates

A total of nine *C. muris* isolates and 43 *C. andersoni* isolates were used in this study (Table 1). The *C. muris* isolates were from Siberian chipmunk, hamsters, and ostriches in Henan province. The *C. andersoni* isolates were from hamsters, sheep, and cattle (including dairy cattle and beef cattle) in Henan, Jilin, Heilongjiang, Shaanxi, Sichuan, and Guangxin provinces. Some of the *C. muris* and *C. andersoni* DNA specimens (*C. muris*: MC2, MC4, MC14, and MC17; *C. andersoni*: MC7, MC16, CL01, SP69, SP75, DY-ZZ7, DY-ZZ8, DY-ZZ13, DY-ZZ17, DY-ZZ30, DY-ZZ31, DY-ZZ47, DY-ZZ48, DY-HLJ3, DY-HLJ9, DY-HLJ13, DY-HLJ14, and DY-HLJ18) (Table 1) are part of our laboratory’s archive, which have been identified in previous studies [7], [16], [35], [36], whereas the remaining isolates were diagnosed as positive for *C. muris* or *C. andersoni* by PCR-RFLP and DNA sequence analysis of a ∼830 bp fragment of the small subunit (SSU) rRNA gene [37].

**Table 1:** Isolates used in this study and their subtype identity at the four minisatellite loci.

Isolate ID	Species	Host	Geographic source	MLST subtype			
				MS1	MS2	MS3	MS16
MC2	*C. muris*	Siberian chipmunk	Henan	M11	M4	M6	M1
MC4	*C. muris*	Hamster	Henan	M11	M4	M6	M1
MC14	*C. muris*	Hamster	Henan	M11	M4	M6	M1
MC17	*C. muris*	Hamster	Henan	M11	M4	M6	M1
OH1	*C. muris*	Ostrich	Henan	M5	M4	M6	M4
OH2	*C. muris*	Ostrich	Henan	M5	M4	M6	M4
OH14	*C. muris*	Ostrich	Henan	M5	M4	M6	M4
OH16	*C. muris*	Ostrich	Henan	M5	M4	M6	M4
OH18	*C. muris*	Ostrich	Henan	M5	M4	M6	M4
MC7	*C. andersoni*	Hamster	Henan	A3	A4	A2	A2
MC16	*C. andersoni*	Hamster	Henan	A3	A4	A2	A2
CL01	*C. andersoni*	Bactrian camel	Henan	A6	A5	A2	A1
CL02	*C. andersoni*	Bactrian camel	Henan	A6	A4	A2	A1
SP69	*C. andersoni*	Sheep	Henan	A2	A5	A2	A1
SP75	*C. andersoni*	Sheep	Henan	A2	A4	A2	A1
DY-LB2	*C. andersoni*	Dairy cattle	Henan	A4	A4	A4	A1
DY-LB8	*C. andersoni*	Dairy cattle	Henan	A4	A4	A4	A1
DY-LY2	*C. andersoni*	Dairy cattle	Henan	A4	A4	A4	A1
DY-LY3	*C. andersoni*	Dairy cattle	Henan	A4	A4	A4	A1
DY-ZZ7	*C. andersoni*	Dairy cattle	Henan	A2	A4	A2	A1
DY-ZZ8	*C. andersoni*	Dairy cattle	Henan	A4	A4	A4	A1
DY-ZZ13	*C. andersoni*	Dairy cattle	Henan	A4	A4	A4	A1
DY-ZZ17	*C. andersoni*	Dairy cattle	Henan	A3	A4	A4	A1
DY-ZZ30	*C. andersoni*	Dairy cattle	Henan	A4	A4	A4	A1
DY-ZZ31	*C. andersoni*	Dairy cattle	Henan	A1	A4	A4	A1
DY-ZZ47	*C. andersoni*	Dairy cattle	Henan	A2	A4	A2	A1
DY-ZZ48	*C. andersoni*	Dairy cattle	Henan	A4	A4	A4	A1
DY-HLJ3	*C. andersoni*	Dairy cattle	Heilongjiang	A4	A4	A4	A1
DY-HLJ9	*C. andersoni*	Dairy cattle	Heilongjiang	A4	A4	A4	A1
DY-HLJ13	*C. andersoni*	Dairy cattle	Heilongjiang	A4	A4	A4	A1
DY-HLJ14	*C. andersoni*	Dairy cattle	Heilongjiang	A4	A4	A4	A1
DY-HLJ18	*C. andersoni*	Dairy cattle	Heilongjiang	A4	A4	A4	A1
DY-JL6-3	*C. andersoni*	Dairy cattle	Jilin	Noisy	–	A4	–
DY-JL26	*C. andersoni*	Dairy cattle	Jilin	A4	A4	A4	A1
DY-SC1	*C. andersoni*	Dairy cattle	Sichuan	A4	A4	A4	A1
DY-SC2	*C. andersoni*	Dairy cattle	Sichuan	A4	A4	A4	A1
DY-SC3	*C. andersoni*	Dairy cattle	Sichuan	A2	A4	A4	A1
DY-SC5	*C. andersoni*	Dairy cattle	Sichuan	A1	A4	A4	A1
DY-SC6	*C. andersoni*	Dairy cattle	Sichuan	A4	A4	A4	A1
DY-GX1	*C. andersoni*	Dairy cattle	Guangxi	A1	A4	A4	A1
DY-GX5	*C. andersoni*	Dairy cattle	Guangxi	A1	A4	A4	A1
DY-GX6	*C. andersoni*	Dairy cattle	Guangxi	A2	A4	A2	A1
DY-GX7	*C. andersoni*	Dairy cattle	Guangxi	A4	A4	A4	A1
DY-SX96	*C. andersoni*	Dairy cattle	Shanxi	A4	A4	A4	A1
BF-SX00	*C. andersoni*	Beef cattle	Shanxi	A4	A4	A4	A1
BF-SX13	*C. andersoni*	Beef cattle	Shanxi	A4	A4	A4	A1
BF-SX23	*C. andersoni*	Beef cattle	Shanxi	A4	A4	A4	A1
BF-SX101	*C. andersoni*	Beef cattle	Shanxi	A4	A4	A4	A1
BF39	*C. andersoni*	Beef cattle	Henan	A4	A4	A4	A1
BF43	*C. andersoni*	Beef cattle	Henan	A5	A4	A4	A1
BF156	*C. andersoni*	Beef cattle	Henan	A1	A4	A4	A1
BF160	*C. andersoni*	Beef cattle	Henan	A1	A4	A4	A1

### DNA Extraction and Subtyping

Genomic DNA was extracted from *Cryptosporidium*-positive feces samples using the E.Z.N.A.® Stool DNA kit (Omega Biotek Inc., Norcross, USA) and the manufacturer-recommended procedures. Primers and amplification conditions used in nested-PCR analysis of MS1 (coding for hypothetical protein), MS2 (coding for 90 kDa heat shock protein), MS3 (coding for hypothetical protein), and MS16 (coding for leucine rich repeat family protein) genes were previously described [31]. KOD-Plus-Neo amplification enzyme (Toyobo Co. Ltd, Osaka, Japan) was used for PCR amplification. 400 ng/µl of non-acetylated bovine serum albumin (Solarbio Co. Ltd, Beijing, China) was used in the primary PCR to neutralize PCR inhibitors. The secondary PCR products were examined by agarose gel electrophoresis and visualized after GelRed™ (Biotium Inc., Hayward, CA) staining. The secondary PCR products were sequenced on an ABI 3730 DNA Analyzer (Applied Biosystems, Foster City, USA), using the secondary primers and the Big Dye Terminator v3.1 Cycle Sequencing kit (Applied Biosystems). The sequence accuracy was confirmed by two-directional sequencing and by sequencing a new PCR product if necessary.

### Data Analysis

Sequence alignment was done using the program ClustalX 1.83 (ftp://ftp-igbmc.u-strasbg.fr/pub/ClustalX/). Neighbor-joining trees were constructed using the program Phylip version 3.69, based on the evolutionary distances calculated by Kimura-2-parameter model. DnaSP version 5.10.01 (http://www.ub.edu/dnasp/) was used to analyze the genetic diversity of the *C. muris* and *C. andersoni* sequences. Linkage disequilibrium across all loci was assessed using the standardized index of association (*I* ^S^_A_) proposed by Habould and Hudson [38]. The index and its probability under a null model of complete panmixia were calculated using LIAN version 3.5 (http://adenine.biz.fh-weihenstephan.de/cgi-bin/lian/lian.cgi.pl) with hypothesis testing by a parametric method. The genetic structures of *C. muris* and *C. andersoni* groups were calculated using STRUCTURE version 2.3.3 by *K*-means partitional clustering and the admixture model. STRUCTURE calculated membership coefficients to place all the individuals to *K* clusters, where *K* value was set from 2 to 8 in this study and the most appropriate number of *K* was determined by calculating delta *K* as described in a previous study [39].

### Nucleotide Sequence Accession Numbers

Representative nucleotide sequences were deposited in the GenBank under accession numbers JF732833 to JF732872.

## Results

### Subtypes of *C. muris* and *C. andersoni*

**Figure 1:**
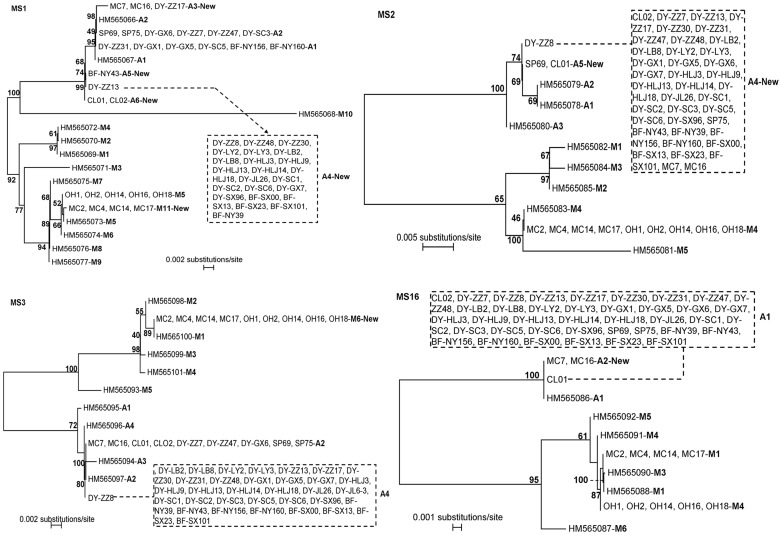
Phylogenetic relationship among subtypes of *C. muris* and *C. andersoni* at four microsatellite and minisatellite loci (MS1, MS2, MS3, and MS16) as assessed by a neighbor-joining analysis of the nucleotide sequences, using distance calculated by the Kimura 2-parameter model.

A total of 52 isolates were successfully subtyped at all four loci. In contrast, only MS1 and MS3 were amplified for isolate DY-JL6-3. At each of the four loci, the acquired sequences consisted of two groups by multiple-sequence alignment analysis: one group consisted of *C. muris* isolates and the second one was all *C. andersoni* isolates. This was supported by results of phylogenetic analysis (Figure 1). Altogether, 2, 1, 1, and 2 subtypes were identified in *C. muris*, and 6, 2, 2, and 2 subtypes in *C. andersoni* at the MS1, MS2, MS3, and MS16 loci, respectively (Figure 1). Among them, two *C. muris* subtypes and seven *C. andersoni* subtypes represented new subtypes (Figure 1).

### Nature of Polymorphism in Minisatellite Sequences

The two groups of parasites identified differed from each other by having numerous nucleotide substitutions in the non-repeat region. Within each group, sequences differed from each other only in the number of minisatellite repeats. The insertions and deletions were always in trinucleotides because of the coding nature of the targets.

The two species differed from each other in the nature of minisatellite repeats at some loci. At the MS16 locus, *C. muris* and *C. andersoni* had the same repeat sequence (CTTCTTCAT). However, the repeat sequences of *C. muris* and *C. andersoni* differed from each other at the MS2 and MS3 loci. In addition, the extent of differences in repeat sequences also varied by locus. At the MS1 locus, only one nucleotide difference was noticed in one of the two minisatellite regions between *C. muris* and *C. andersoni*. In contrast, the repeat sequences were totally different at the MS3 locus (Table 2).

**Table 2:** The nature of minisatellite repeats at four genetic loci.

Locus	*C. muris*	*C. andersoni*
MS1	(TAAAGGGAGAGA)_3_ & (GAACGAGATAGG)_15,18_	(TAAAGGGCGAGA)_3_ & (GAACGAGATAGG)_12–17_
MS2	(CCATATCCC)_3_ & (CCATACCTC)_3_	(CCATACCTC)_10–11_
MS3	(TGTTGG)_10_ & (GCTGCA)_6_	(TGTTGGTGTTGCTGT)_2_ & (TGCTGCAGCTGC)_2–3_
MS16	(CTTCTTCAT)_10–11_	(CTTCTTCAT)_12,14_

### Multilocus Subtypes and Polymorphism

Except for a *C. andersoni* isolate (DY-JL6-3), the remaining isolates were successfully subtyped at all four loci, forming two *C. muris* and 10 *C. andersoni* MLST subtypes. Three *C. muris* isolates from Siberian chipmunk and hamsters, and five *C. muris* isolates from ostriches each formed a single MLST subtype. In *C. andersoni*, the MLST subtype A4, A4, A4, A1 had the most number of isolates (n = 24), followed by MLST subtype A1, A4, A4, A1 (n = 6). In contrast, other eight MLST subtypes had 1–4 isolates (Table 1). Thus, a total of 14 *C. muris* and 17 *C. andersoni* MLST subtypes have been identified, including those reported previously [31] (Table S1).

Sequence data of all four loci, including the data reported by Feng et al. [31], were concatenated making a multilocus gene of 2056 bp length for *C. muris* and 2142 bp length for *C. andersoni*. Genetic diversity of sequences was analyzed using DnaSP version 5.10.01. The former produced 59 polymorphic sites and 4 haplotypes with a haplotype diversity of 0.677±0.075, nucleotide diversity of 0.00734, and average number of nucleotide differences of 14.24 (Table 3). The latter had 4 polymorphic sites and 5 haplotypes with a haplotype diversity of 0.384±0.079, nucleotide diversity of 0.00024, and average number of nucleotide differences of 0.477 (Table 3).

**Table 3:** Genetic diversity of *C. andersoni* and *C. muris* DNA sequences.

*Cryptosporidium* species	Number of sequences	Number of sites	No. of polymorphicsites, S	No. of haplotypes, h	Haplotype diversity, Hd	Nucleotide diversity, Pi	Average number of nucleotide differences, k
*C. muris*	25	2056	59	4	0.677±0.075	0.00734	14.24
*C. andersoni*	54	2142	4	5	0.384±0.079	0.00024	0.477

### Linkage Disequilibrium Analysis

The *I* ^S^_A_ values for the populations are shown in Table 4. When all isolates were used in the analysis, the *C. muris* and *C. andersoni* populations both had positive *I* ^S^_A_ values and the pairwise variance (*V*_D_) was greater than the 95% critical value (*L*) indicating the presence of linkage disequilibrium (LD) in both populations. To test for the possibility that LD could be due to clonal expansion of one or more subtypes which masks the underlying equilibrium, *I* ^S^_A_ was calculated for MLST subtypes only (considering each group of isolates with the same MLST subtype as one individual) for *C. muris* and *C. andersoni*. The *I* ^S^_A_ value obtained was still above zero in the *C. muris* population (*I* ^S^_A_ = 0.1355, *V*_D_ >*L*). In contrast, negative values (−0.0094 and −0.0109) of *I* ^S^_A_ were obtained for the *C. andersoni* population from various animals. The same analysis was performed for *C. andersoni* in cattle in China, which suggested that this population had an epidemic population structure (*I* ^S^_A_ = 0.0290, *V*_D_ <*L*).

**Table 4:** Analysis of linkage disequilibrium in *C. andersoni* and *C. muris* populations.

species	Area	Source of isolates	No. ofcompletelytyped	Standardizedindex ofassociation (*I* ^S^_A_)	*P* value	*V*_D_ >*L*
*C. andersoni*	China, USA, Australia, Czech	Cattle, bactrian camel, sheep, hamster	54	0.2058	1.85×10^−23^	Yes
	China	Cattle, bactrian camel, sheep, hamster	48	0.2520	3.71×10^−25^	Yes
	China	Cattle, bactrian camel, sheep	46	0.2542	1.86×10^−28^	Yes
	China	Cattle	42	0.2422	3.04×10^−19^	Yes
	China, USA, Australia, Czech	Cattle, bactrian camel, sheep, hamster	17^a^	−0.0094	1	No
	China	Cattle, bactrian camel, sheep, hamster	13^a^	−0.0109	1	No
	China	Cattle	9^a^	0.0290	6.14×10^−1^	No
*C. muris*	China, Czech, Kenya, Egypt	Bactrian camel, human, mara (*Dolichotis patagonum*),bactrian camel via *Mastomys coucha*, mountaingoat, laboratory mouse, mouse, Yellow ratsnake, ostrich, Siberian chipmunk, hamster, squirrel,Tachyorectes via *Meriones unguiculatus*, camel viamice, dog via mice, RN66 via SCID mice	26	0.3288	6.45×10^−73^	Yes
	China, Czech, Kenya, Egypt	Bactrian camel, human, mara (*Dolichotis patagonum*),bactrian camel via *Mastomys coucha*, mountaingoat, laboratory mouse, mouse, Yellow rat snake,ostrich, Siberian chipmunk, hamster, squirrel,Tachyorectes via *Meriones unguiculatus*, camelvia mice, dog via mice, RN66 via SCID mice	14^a^	0.1093	2.79×10^−4^	Yes

**Note**: *V*_D_ = the pairwise variance, *L = *95% critical value; ^a^with the same MLST type as one individual; the data of the non-Chinese isolates came from a recently published paper [^31^].

### Population Substructure

**Figure 2:**
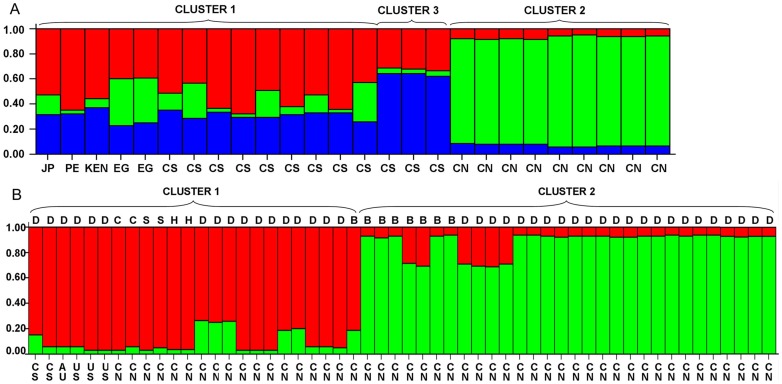
Population structure inferred by Bayesian clustering using multilocus subtype information. A, *Cryptosporidium muris*; B, *Cryptosporidium andersoni*. Each individual is shown as a thin vertical line, which is partitioned into *K* colored components representing estimated membership fractions in *K* genetic clusters, and the geographic locations are at the bottom. The pie charts show the distribution of genetic clusters in different countries and various animals. JP = Japan; PE = Peru; Ken = Kenya; EG = Egypt; CS = Czech Republic; CN = China; B = beef cattle; C = bactrian camel; D = dairy cattle; H = hamster; S = sheep.

A Bayesian statistical approach was used to infer population substructure in allelic variation in the minisatellite sequences using the software STRUCTURE. The peak value of delta *K* was noticed at *K* = 3, thus, *Cryptosporidium muris* produced 3 clusters (Figure 2A). Cluster 2 consisted of the *C. muris* samples from hamsters and ostriches in China, Cluster 3 contained three laboratory passaged *C. muris* isolates from the Czech Republic, including bactrian camel via *Mastomys coucha*, RN66 via SCID mice, and *Tachyorectes* via *Meriones unguiculatus*, while cluster 1 included the remaining *C. muris* isolates from Japan, Peru, Kenya, Egypt, and Czech Republic (Figure 2A). Likewise, two clusters (*K* = 2) were identified in *C. andersoni* isolates. Cluster 1 included isolates from dairy cattle in the United States, Czech Republic, and Australia, and bactrian camel, sheep, hamster, and a small number of dairy cattle and beef cattle in China. In contrast, cluster 2 consisted of most *C. andersoni* isolates from dairy cattle and beef cattle in China (Figure 2B).

## Discussion

In this study, 1–2 subtypes of *C. muris* and 2–6 subtypes of *C. andersoni* were seen at each of the polymorphic loci. The sequence polymorphism in *C. muris* and *C. andersoni* was largely in the form of differences in the copy number of minisatellite repeats (Table 2). Thus, as discussed in a more recent study [31], the coding nature of the targets was probably not responsible for the differences observed between the gastric and intestinal *Cryptosporidium* spp. In contrast, this difference might be a reflection of intrinsic biologic and genetic difference between gastric and intestinal *Cryptosporidium* species.

Multilocus DNA sequence analysis by DnaSP showed that the genetic diversity of *C. andersoni* was much smaller than that of *C. muris* (Table 3), which might attribute to the narrow host specificity of *C. andersoni* [31]. For both parasites, genetic differences were observed depending on the animal host species. For example, the MLST subtype of *C. muris* in ostriches obviously differed from those in bactrian camel, mice, squirrels, dogs, mountain goats, maras, and humans (Table 1) [31]. Likewise, differences were also noticed in the MLST subtypes of *C. andersoni* among hamsters, bactrian camels, sheep, and cattle (Table 1). These differences observed may be a reflection of co-evolution of hosts and parasites, which might lead to different biologic characteristics. For example, *C. andersoni* isolates in Japan, the so-called Kawatabi strain, differ from *C. andersoni* isolates in other areas in its ability to infect SCID mice [40].

In the present study, the *I* ^S^_A_ values for *C. muris* and *C. andersoni* populations were all above zero when all isolates from various animals were included in the analysis (Table 4), which indicated both *C. muris* and *C. andersoni* populations had clonal genetic structure and genetic exchange occurred rarely. Therefore, unlike *Cryptosporidium parvum*, the number of subtypes of *C. muris* and *C. andersoni* was relatively less. When each group of isolates with the same MLST subtype was considered as one individual, data analysis showed that LD still existed in *C. muris* population (*I* ^S^_A_ = 0.1355, *V*_D_ > *L*). Conversely, although “statistically significant," the *I* ^S^_A_ value for *C. andersoni* isolates was near zero (Table 4), suggesting it could not be the evidence for panmictic population structure. Interestingly, the same analysis indicated that the *C. andersoni* in cattle in China had an epidemic population structure (*I* ^S^_A_ = 0.0290, *V*_D_ <*L*). These results, combining with different MLST subtypes compared to other countries, suggested that the prevalence of *C. andersoni* in China is not attributed to the introduction of dairy cattle based on the following facts: 1) the introduction of dairy cattle in China only occurred in the last 20 years and the main breed is Holstein cattle from Australia and New Zealand [41]; 2) *Cryptosporidium andersoni* was present in China in non-dairy areas and before the introduction of Holstein cattle [42] and 3) the *C. parvum* IId subtype (IIdA19G1) found in cattle in China has not been reported in cattle in Australia and New Zealand, or most other places in the world [36]. Thus, diverse factors including transmission dynamics, geographical isolation, and host-specificity might contribute to the emergence of epidemic populations.

STRUCTURE analysis showed that the *Cryptosporidium muris* population formed three clusters (Figure 2A). Among which, three “*C. muris* variant" isolates from the Czech Republic including an isolate (TS03) originated from East African mole rat (*Tachyoryctes splendens*) formed a single substructure. This result was in agreement with previous observations that the East African isolate differed from other *C. muris* isolates based on cross-transmission, genotyping and subtyping studies [30], [43]. In addition, Chinese *C. muris* isolates from rodents and ostriches also consisted of a separate cluster. Thus, the substructure of *C. muris* noticed in this study further confirmed the existence of genetic and biologic diversity in *C. muris*.

*Cryptosporidium andersoni* formed two clusters in the STRUCTURE analysis. Most *C. andersoni* isolates from dairy cattle and beef cattle in China belonged to a separate cluster, whereas the *C. andersoni* isolates from other animals formed a different cluster. Therefore, as discussed above, this observation provides further evidence that the prevalence of *C. andersoni* in China is not attributed to the introduction of dairy cattle. On the other hand, cluster 2 consisted of the MLST subtypes (A4, A4, A4, A1) (n = 24) and (A1, A4, A4, A1) (n = 6) (Figure 2B), which represented the two most common subtypes found in cattle in China. Thus, the clonal expansion of such subtype might have led to the epidemic population structure of *C. andersoni* in cattle in China.

In conclusion, as expected, multiple MLST subtypes of *C. muris* or *C. andersoni* were present in various animals examined in the present study. The *C. muris* and *C. andersoni* populations examined in this and a previous study had an overall clonal genetic structure, with the Chinese *C. andersoni* population in cattle having an epidemic structure. Georgaphic isolation and host-adaptation were both observed in *C. muris* and *C. andersoni* populations. In addition, the present study suggested that the prevalence of *C. andersoni* in China is not attributed to the introduction of dairy cattle. Nevertheless, more studies are needed to better elucidate the genetic basis for the difference in host specificity in the two most common gastric parasites, and the population genetic structure and spread of *C. muris* and *C. andersoni* in the world.

## Supporting Information

Table S1 **The frequency and allele composition of the multilocus sequence types including those reported by Feng et al.** [**^31^**]
(DOC)
