# Supplementary material for: Multilocus Sequence Subtyping and Genetic Structure of Cryptosporidium muris and Cryptosporidium andersoni
Source: PLoS One. 2012 Aug 24;7(8):e43782. doi: 10.1371/journal.pone.0043782 (PMC3427161; doi:10.1371/journal.pone.0043782)
Supplement: Table S1 — The frequency and allele composition of the multilocus sequence types including those reported by Feng et al. [31] (DOC) [file pone.0043782.s001.doc]

**Table S1** The frequency and allele composition of the multilocus sequence types including those reported by Feng et al. [31]

| **Multilocus sequence type** | **Allele composition** | | | | **No. of isolates** |
| --- | --- | --- | --- | --- | --- |
|  | **MS1** | **MS2** | **MS3** | **MS16** |  |
| ***C. andersoni*** |  |  |  |  |  |
| 1 | 6 | 5 | 2 | 1 | 1 |
| 2 | 6 | 4 | 2 | 1 | 1 |
| 3 | 2 | 5 | 2 | 1 | 1 |
| 4 | 3 | 4 | 4 | 1 | 1 |
| 5 | 2 | 4 | 4 | 1 | 1 |
| 6 | 5 | 4 | 4 | 1 | 1 |
| 7 | 3 | 4 | 2 | 2 | 2 |
| 8 | 2 | 4 | 2 | 1 | 4 |
| 9 | 1 | 4 | 4 | 1 | 6 |
| 10 | 4 | 4 | 4 | 1 | 24 |
| 11 | 1 | 2 | 4 | 1 | 3 |
| 12 | 2 | 1 | 3 | 1 | 1 |
| 13 | 2 | 1 | 2 | 1 | 2 |
| 14 | 1 | 3 | 4 | 1 | 1 |
| 15 | 2 | 3 | 4 | 1 | 3 |
| 16 | 2 | 3 | 1 | 1 | 1 |
| 17 | 2 | 3 | 2 | 1 | 1 |
| ***C. muris*** |  |  |  |  |  |
| 1 | 5 | 4 | 1 | 5 | 1 |
| 2 | 1 | 2 | 4 | 5 | 2 |
| 3 | 6 | 4 | 1 | 2 | 1 |
| 4 | 5 | 4 | 2 | 3 | 2 |
| 5 | 7 | 4 | 2 | 1 | 1 |
| 6 | 7 | 4 | 1 | 5 | 1 |
| 7 | 9 | 4 | 1 | 1 | 2 |
| 8 | 1 | 1 | 4 | 5 | 1 |
| 9 | 8 | 4 | 2 | 4 | 1 |
| 10 | 10 | 5 | 5 | 6 | 3 |
| 11 | 6 | 4 | 2 | 4 | 1 |
| 12 | 2 | 2 | 3 | 5 | 1 |
| 13 | 11 | 4 | 6 | 1 | 4 |
| 14 | 5 | 4 | 6 | 4 | 5 |
